# Antibody Levels to Persistent Pathogens and Incident Stroke in Mexican Americans

**DOI:** 10.1371/journal.pone.0065959

**Published:** 2013-06-14

**Authors:** Shawnita Sealy-Jefferson, Brenda W. Gillespie, Allison E. Aiello, Mary N. Haan, Lewis B. Morgenstern, Lynda D. Lisabeth

**Affiliations:** 1 Department of Family Medicine and Public Health Sciences, Wayne State University School of Medicine, Detroit, Michigan, United States of America; 2 Department of Biostatistics, University of Michigan, School of Public Health, Ann Arbor, Michigan, United States of America; 3 Center for Statistical Consultation and Research, University of Michigan, Ann Arbor, Michigan, United States of America; 4 Department of Epidemiology, Center for Social Epidemiology and Population Health, University of Michigan, School of Public Health, Ann Arbor, Michigan, United States of America; 5 Department of Epidemiology and Biostatistics, University of California, San Francisco, California, United States of America; 6 Stroke Program, University of Michigan Medical School, Ann Arbor, Michigan, United States of America; 7 Department of Epidemiology, University of Michigan, School of Public Health, Ann Arbor, Michigan, United States of America; Auburn University, United States of America

## Abstract

**Background:**

Persistent pathogens have been proposed as risk factors for stroke; however, the evidence remains inconclusive. Mexican Americans have an increased risk of stroke especially at younger ages, as well as a higher prevalence of infections caused by several persistent pathogens.

**Methodology/Principal:**

Findings Using data from the Sacramento Area Latino Study on Aging (n = 1621), the authors used discrete-time regression to examine associations between stroke risk and (1) immunoglobulin G antibody levels to *Helicobacter pylori (H. pylori*), Cytomegalovirus, Varicella Zoster Virus, *Toxoplasma gondii* and Herpes simplex virus 1, and (2) concurrent exposure to several pathogens (pathogen burden), defined as: (a) summed sero-positivity, (b) number of pathogens eliciting high antibody levels, and (c) average antibody level. Models were adjusted for socio-demographics and stroke risk factors. Antibody levels to *H. pylori* predicted incident stroke in fully adjusted models (Odds Ratio: 1.58; 95% Confidence Interval: 1.09, 2.28). No significant associations were found between stroke risk and antibody levels to the other four pathogens. No associations were found for pathogen burden and incident stroke in fully adjusted models.

**Conclusions/Significance:**

Our results suggest that exposure to *H. pylori* may be a stroke risk factor in Mexican Americans and may contribute to ethnic differences in stroke risk given the increased prevalence of exposure to *H. pylori* in this population. Future studies are needed to confirm this association.

## Introduction

In the United States, stroke is a significant public health issue, affecting roughly 795,000 individuals annually [1]. Mexican Americans (MAs) are among the fastest growing populations in the United States [2], and stroke incidence is higher in MAs than in Non-Hispanic whites (NHWs), especially at younger ages [3]. Aside from diabetes [4], large ethnic differences in the prevalence of stroke risk factors do not exist, which suggests that traditional stroke risk factors are unlikely to fully account for the ethnic disparity in stroke risk. MAs have a higher prevalence of infections caused by several persistent pathogens that have been linked to chronic diseases, including Cytomegalovirus (CMV) [5], *Helicobacter pylori* (*H. pylori*) [6], *Toxoplasma gondii* (*T. gondii*) [7], and Herpes simplex virus 2 (HSV2) [8]. If persistent pathogen exposure is a risk factor for stroke, this exposure may contribute to the ethnic disparity in stroke risk.

Many persistent pathogens are never completely cleared from the host, and as a result, are prone to reactivation which could lead to the promotion and/or worsening of atherosclerosis [9], [10], which is the major underlying pathophysiology of ischemic stroke. [11] Aside from exposure to individual pathogens, total pathogen burden, or exposure to multiple pathogens concurrently, may impact stroke risk by invoking a stronger immune system response than what would be apparent with exposure to a single persistent infection. Persistent pathogens, traditional risk factors, and genetic predisposition may work together in stimulating inflammatory pathways that promote atherosclerosis, which in turn may increase stroke risk [12].

To date, prospective studies have not identified links between individual persistent pathogens and incident stroke. Investigators using data from the Framingham Heart Study (n = 1,187) reported no association between sero-positivity to Chlamydia pneumoniae (C. pneumoniae), *H. pylori* and CMV infections and incident stroke [13]. Elkind et al. also reported no significant association between sero-positivity to five infectious pathogens (C. pneumoniae, *H. pylori*, CMV, HSV-1 and HSV-2) and stroke risk, in a multi-ethnic prospective cohort (n = 1625) with an 8 year (median) follow-up [14]. Most recently, in a five year prospective cohort study, no significant association was found between sero-positivity to cytotoxin associated A-gene negative or positive strains of *H. pylori* and stroke risk [15].

The effect of pathogen burden on stroke risk has not been well studied. Elkind et. al reported a positive association between an infectious disease burden index, that included several pathogens, and risk of stroke after multivariable adjustment (Hazard Ratio (HR):1.39; 95% CI: 1.02, 1.90) [14] which supports the hypothesis that pathogen burden may be a more important stroke risk factor than is exposure to individual pathogens. However, other studies, including Framingham, have not found an association between pathogen burden and stroke [13], [16].

Importantly, the existing studies investigating the link between persistent pathogens and stroke risk were not focused on the MA population [3], [5], [6], [7], [8]. Further, these studies were limited by their use of sero-positivity, which is a dichotomous indicator of pathogen exposure, instead of antibody level to infection, which may be a more consistent predictor of inflammatory outcomes [17]. Given these limitations, our objective was to examine the associations between incident stroke and: (1) antibody levels to five individual persistent pathogens including previously studied pathogens (CMV, HSV1, and *H. pylori*), as well as two novel pathogens (VZV and *T. gondii*) given their ability to persist latently and reactivate, and produce a chronic inflammatory response, and (2) pathogen burden operationalized in several novel ways utilizing antibody levels, in an elderly cohort of MAs from the Sacramento Area Latino Study on Aging (SALSA).

## Methods

### Ethics Statement

The University of Michigan Institutional Review Board approved this study HUM00042648.

### Study Population

SALSA is a longitudinal study of community-dwelling Latinos residing in the Sacramento Valley of California. At baseline (1998–1999), 1,789 Latino participants who were 60–101 years of age were enrolled. Details of the recruitment and enumeration process, as well as survey design, have been published previously [18], [19]. Over the course of the 10 year study period, total attrition from deaths (n = 452), refusals (n = 169) and loss to follow up (n = 147) in the cohort averaged 5.4% per year. Attrition due to deaths averaged 3% per year, refusals 1.2% and loss to follow-up 1%. We excluded 168 participants who had a self-reported history of stroke at baseline, and as a result, our analysis sub-sample included 1,621 study subjects.

Study participants underwent a baseline in-home interview and medical exam, where a trained bilingual interviewer collected a fasting serum sample, data on socio-demographics, medical history, medication usage, behavioral risk factors, and clinical, cognitive and functional status. Seven in-home interviews were conducted at 12–15 month intervals, to update the information collected during the baseline interview. Fasting serum samples were collected at baseline and during the 3–6^th^ annual in-home interviews. Study subjects also participated in semi-annual telephone interviews (n = 6), where information on medical history, medication usage, and demographics was collected. This study was approved by the Institutional Review Boards at the University of Michigan, and the University of California, San Francisco and Davis, and all study subjects gave written consent.

### Laboratory Analyses

Serum samples were tested at the Stanley Neurovirology Laboratory of the Johns Hopkins University School of Medicine for Immunoglobulin G (IgG) antibody levels to *H. pylori*, CMV, HSV1, VZV, and *T. gondii*, measured by optical density ratio units, using a commercially available solid-phase enzyme-linked immunosorbent assay, with instructions from the manufacturers used to determine sero-status. Individuals were categorized as sero-negative to CMV, HSV1, *H. pylori*, and VZV if their absorbance values were ≤0.9; equivocal if between 0.9 and 1.1; and sero-positive if ≥1.1 [20]. The cut-point for sero-positivity to *T. gondii* was 10 international units/ml [20]. For the analysis, individual pathogen variables were defined using sero-status (positive/negative, with equivocal values categorized as negative) and antibody levels, based on IgG antibody levels. Pathogen burden variables were defined as: (1) summed sero-positivity to 5 pathogens (for comparison with previous studies), (2) sum of the number of pathogens with high antibody level, defined as IgG antibody response in the top quartile of the baseline distribution for the pathogen, and (3) average z-scored IgG antibody level across all 5 pathogens, including those individuals sero-positive to at least one pathogen.

### Measures

Incident strokes were identified from study participants using the following methods. At the baseline visit, participants were asked “Has a doctor ever told you that you had a stroke?”. At each subsequent follow-up visit and semi-annual telephone conversation, participants were asked whether a doctor ever told them that they had a stroke or cerebrovascular accident since the last interview. Fatal strokes were identified from death certificates using the international classification of disease-10 code 164. Death certificates were obtained on 90.2% (n = 414) of those reported to be deceased by their families and/or by vital statistics. Incident strokes and stroke deaths confirmed by death certificate were included as outcome events. Self-reported stroke has been shown to accurately estimate incident stroke [21], [22], [23].

Variables included in the analysis as potential confounders included hypertension, diabetes, hyperlipidemia, smoking, atrial fibrillation, body mass index (BMI), coronary heart disease and/or peripheral artery disease (PAD), education, age and gender. Two blood pressure measurements were taken using an automatic digital blood pressure monitor (OMRON MODEL: HEM-747 IC). Individuals were categorized as hypertensive (yes/no) if they self-reported a physician diagnosis of hypertension, reported use of anti-hypertensives, or had a sitting systolic blood pressure of at least 140 mm Hg and/or a diastolic blood pressure of at least 90 mm Hg. Diagnosis of diabetic status (yes/no) was determined by the presence of a serum fasting glucose of >125 mg/dl, self-report of physician diagnosis of diabetes, or use of a diabetic medication. Morning fasting serum samples were used to test for total cholesterol using Reagent for Cholesterol (number 3313018; Roche Diagnostics, Indianapolis, Indiana), low density lipoprotein (LDL) cholesterol using the LDL Direct Liquid Select (number 7120; Equal Diagnostics) and high density lipoprotein (HDL) cholesterol using the HDL Direct Reagent (number 3034569; Roche Diagnostics, Indianapolis, Indiana). Hyperlipidemia (yes/no) was defined as: having both LDL >100 mg/dl and HDL<40 mg/dl or total cholesterol level >200 mg/dl. Smoking status was categorized as ever/never for the analysis since only 11.10% of the study population were current smokers (n = 180). History of atrial fibrillation (yes/no) was determined by self-reported physician diagnosis within the previous 5 years. The body mass index (BMI) variable was defined using height and weight measurements taken during in person interviews, and was modeled continuously. Diagnosis of myocardial infarction, angina pectoris, intermittent claudication, congestive heart failure, and heart/coronary catheterization was determined by self-report of any prior physician diagnosis of these conditions. A coronary heart disease/PAD indicator variable was defined as having a history of at least 1 of the following conditions: myocardial infarction, angina pectoris, intermittent claudication, congestive heart failure, and heart/coronary heart catheterization. Years of education was categorized as 0–3, 4–11 and ≥12.

Baseline descriptive statistics for socio-demographics as well as prevalence of stroke risk factors were calculated and compared by incident stroke status using χ^2^ tests for categorical variables and Wilcoxon rank-sum test for continuous variables. Correlation among the five persistent pathogens was assessed with χ^2^ tests of independence (sero-status) and spearman correlations (antibody levels). Discrete-time logistic regression analyses were performed to investigate associations between exposure to individual persistent pathogens and measures of pathogen burden and incident stroke using data from 13 time points. The discrete-time logistic regression analysis performed here is asymptotically equivalent to a Cox regression as the interval lengths shrink [24]. Even with fairly wide intervals, this method produces very similar results, in practice, to those from Cox models. This method was convenient to apply to the SALSA study data, which had regular follow-up intervals with one record per interval. It also allowed us to easily incorporate time-varying covariates that were assumed constant within an interval but varying from one interval to the next. We used baseline covariate data and updated with longitudinally collected covariate information when available. Since not all of the variables of interest were ascertained at semi-annual visits, we used the previous annual visit data to carry forward information on hypertension, diabetes, hyperlipidemia, BMI, pathogen sero-status and antibody level.

### Statistical Analyses

The number of individuals with missing data for all 5 pathogens at baseline, and at follow-up visits 3–6 was 523, 1,214, 1,193, 1,499 and 1,057, respectively. To justify carrying forward pathogen data from follow-up visits with serum samples to subsequent visits without samples, we assessed agreement between sero-status at baseline and each subsequent follow-up visit, and between each follow-up visit and succeeding visits (Kappa, Gamma, Somers D, Lambda (symmetric and asymmetric), and McNemar’s Test); the same comparisons were made for IgG antibody level to each pathogen (intraclass correlation and coefficient of variation). There was good agreement between previous and subsequent sero-status and antibody level to each pathogen (File S1).

For the discrete time logistic models, antibody levels for each pathogen were modeled continuously. In order to interpret our regression coefficients as the stroke risk for individuals in the 75^th^ versus the 25^th^ percentile of antibody levels, we re-scaled the continuous antibody level variables by dividing by their corresponding interquartile range. Two levels of covariate adjustment were used: (1) age, gender, education, and (2) age, gender, education, diabetes, atrial fibrillation, smoking, BMI, coronary heart disease/PAD, hypertension, and hyperlipidemia. Interactions between the five pathogens were tested in our fully adjusted models. Two-sided P values <0.05, and confidence intervals that did not overlap 1 were considered significant. All statistical procedures were performed using SAS version 9.2 (SAS Institute, Inc., Cary, North Carolina).

## Results

Baseline characteristics of the study population by incident stroke status (n = 1621) are shown in Table 1. During follow-up 164 incident strokes occurred. The mean (Standard deviation (SD)) age of the study participants was 70.4 (7.04), with 41% male. Study participants who experienced an incident stroke during follow-up were more likely to report hypertension and diabetes at baseline (P: 0.0008 and 0.02), respectively). Roughly 47% of the population was born in the United States, with most of the participants completing less than a high school education and earning less than 2,000 dollars per month. Sero-positivity to each individual pathogen was highest for *H. pylori* (66%), HSV1 (63%) and CMV (60%), with the prevalence of both VZV and *T. gondii* falling below 25%. There was no difference in the distribution of sero-positivity to individual pathogens by incident stroke status. Sero-positivity to each pathogen and antibody levels to *H. pylori* were associated with baseline history of stroke (data not shown). We found no correlations between antibody levels to any of the persistent pathogens, except for between CMV and VZV (P: 0.0003).

**Table 1:** Baseline Participant Characteristics by Incident Stroke Status, SALSA, California, 1998–2008.

	No Stroke (n = 1457)	Incident Stroke (n = 164)	
Characteristic	N (%)	N (%)	P
Age (SD)*	70.2 (6.9)	72.6 (7.9)	0.0004
BMI*	29.74 (6.0)	29.85 (5.5)	0.5532
Men	602 (41.3)	71 (43.3)	0.6265
Nativity			0.8955
Mexico	677 (46.5)	71(43.3)	
United States	686 (47.1)	82 (50.0)	
Other	85 (5.8)	10 (6.1)	
Education (years)			0.2878
0–3	464 (31.8)	59 (36.0)	
4–11	552 (37.9)	59 (36.0)	
≥12	432 (.6)	45 (27.4)	
Smoking Status			0.8795
Ever	769 (52.8)	92 (56.1)	
Never	677 (46.5)	71 (43.3)	
Income			0.7760
1,000/month	635 (43.6)	70 (42.7)	
1,000–1999/month	441 (30.3)	54 (32.9)	
≥2,000/month	349 (24.0)	37 (22.6)	
Hypertension	928 (63.7)	126 (76.8)	0.0008
Diabetes	433 (.7)	66 (40.2)	0.0215
Hyperlipidemia	846 (58.1)	92 (56.1)	0.07
Atrial Fibrillation	74 (5.1)	15 (9.2)	0.0901
Coronary Heart Disease/PAD	260 (17.8)	42 (25.6)	0.0532
Sero-positivity			
CMV	882(60.5)	97 (59.2)	0.1889
* H. pylori*	959 (65.8)	105 (64.0)	0.4574
VZV	9 (20.5)	36 (22.0)	0.6192
HSV1	920(63.1)	94 (57.3)	0.3564
* T. gondii*	353 (24.2)	38 (23.2)	0.7506
All 5 pathogens	56 (3.8)	11 (6.7)	0.0807

* Mean(Standard Deviation (SD));BMI: Body mass index; N: number; P: p-value; SALSA: Sacramento Area Latino Study on Aging; CMV: Cytomegalovirus; *H. pylori: Helicobacter Pylori*; VZV: Varicella zoster virus; HSV1: Herpes simplex virus type 1; *T. gondii: Toxoplasma gondii.*

Table 2 shows the baseline distribution of pathogen burden defined as: summed sero-positivity to 1–5 pathogens and sum of the number of pathogens eliciting a high antibody level, by incident stroke status. The percentage of individuals who were concurrently sero-positive to 1–5 pathogens was similar among those who did and did not have an incident stroke during follow-up (P: 0.49). In terms of the sum of the number of pathogens eliciting a high antibody level by incident stroke status, we found no difference with incident stroke status (P: 0.20), and 16.7% of the study participants had zero pathogens eliciting a high antibody level. The majority of the study population had high antibody levels to only one pathogen (30.8%), followed by those individuals with two (15.9%), three (7.2%) and four or five (1.2%) pathogens concurrently eliciting a high antibody level.

**Table 2:** Baseline Pathogen Burden Defined as Summed Sero-positivity and Number of Pathogens With High IgG Antibody Level, by Incident Stroke Status, SALSA, California, 1998–2008.

	No Stroke (n = 1457)	Incident Stroke (n = 164)	
	N (%)	N (%)	P
**Summed Sero-positivity**			0.4942
Missing	405 (27.8)	52 (31.7)	
0 pathogens	17 (1.2)	2 (1.2)	
1 pathogen	27 (1.9)	2 (1.2)	
2 pathogens	144 (9.9)	16 (9.8)	
3 pathogens	440 (30.2)	47 (28.7)	
4 pathogens	368 (25.3)	34 (20.7)	
5 pathogens	56 (3.8)	11 (6.7)	
**Number with High IgG Antibody Level**			
Missing	405 (27.8)	52 (31.7)	0.1957
0 pathogens	243 (16.7)	28 (17.1)	
1 pathogen	458 (31.4)	41 (25)	
2 pathogens	224 (15.4)	34 (20.7)	
3 pathogens	108 (7.4)	8 (4.9)	
4 or 5 pathogens	19 (1.3)	1 (0.6)	

N: number; P: p-value; SALSA: Sacramento Area Latino Study on Aging.

The associations between IgG antibody levels to the 5 pathogens and stroke risk are shown in Table 3. There were no associations between antibody levels to CMV, HSV1, *T. gondii* or VZV and stroke risk before or after adjustment for covariates. However, *H. pylori* antibody levels were associated with risk of stroke (OR comparing IgG in the 75^th^ versus 25^th^ percentile: 1.58; 95% CI: 1.09, 2.28).

**Table 3:** IgG Antibody Levels to Persistent Pathogens (Comparing 75^th^ and 25^th^ Percentiles) and Incident Stroke, SALSA, California, 1998–2008.

	CMV		HSV1		*T. gondii*		VZV		*H. pylori*	
	OR	95% CI	OR	95% CI	OR	95% CI	OR	95% CI	OR	95% CI
**Model 1** a	0.97	0.76, 1.23	0.93	0.73, 1.18	1.05	0.88, 1.26	1.04	0.86, 1.26	1.27	0.94, 1.72
**Model 2** b	0.85	0.65, 1.12	0.92	0.71, 1.19	1.10	0.91, 1.33	0.95	0.76, 1.19	1.38	1.00, 1.91
**Model 3** c	0.81	0.58, 1.12	0.77	0.56, 1.07	1.03	0.82, 1.29	0.93	0.71, 1.20	1.58	1.09, 2.28

CI: confidence interval; CMV: Cytomegalovirus; *H. pylori*: *Helicobacter pylori*; HSV1: Herpes simplex virus 1; OR: odds ratio; SALSA: Sacramento Area Latino Study on Aging; *T. gondii*: *Toxoplasma gondii*; VZV: Varicella zoster virus;

a Model 1: unadjusted;

b Model 2: age, gender, education;

c Model 3: age, gender, education, diabetes, atrial fibrillation, smoking, coronary heart disease/PAD, hypertension, BMI, and hyperlipidemia.

We performed a post-hoc sensitivity analysis of the association between high antibody levels to *H. pylori* and incident stroke, limiting the analysis to individuals who at baseline, were sero-positive to *H. pylori* (n = 959), and found similar trends to those reported in Table 3 (OR: 1.53; 95% CI: 1.03, 2.28). The association between *H. pylori* and incident stroke was not statistically significantly modified by antibody levels to CMV, HSV-1, VZV, or *T. gondii* (data not shown). In terms of sero-positivity to each individual persistent pathogen, no significant associations with stroke risk were found (data not shown).

Table 4 shows associations between pathogen burden, defined three different ways, and stroke risk. We observed positive associations between incident stroke and both summed sero-positivity to 0–5 pathogens as well as average antibody levels, however, the associations were not significant before or after multivariable adjustment. We found a positive association between sum of the number of pathogens eliciting high antibody response and stroke risk in unadjusted models (OR: 1.18, 95% CI: 1.04, 1.35), however, attenuation and loss of statistical significance occurred after covariate adjustment.

**Table 4:** Pathogen Burden Operationalized Three Different Ways and Incident Stroke, SALSA (n = 1621), California, 1998–2008.

	Model 1^a^		Model 2^b^		Model 3^c^	
Pathogen Burden	OR	95% CI	OR	95% CI	OR	95% CI
Summed Sero-positivity (0–5)	1.10	0.91, 1.33	1.04	0.85, 1.27	1.00	0.80, 1.27
Number of Pathogens Eliciting High Antibody Response (0–5)	1.18	1.04, 1.35	1.11	0.96, 1.30	1.04	0.87, 1.25
Average IgG Antibody Levels	1.19	0.90, 1.57	1.07	0.78, 1.46	0.86	0.58, 1.26

CI: Confidence Interval; OR: Odds Ratio; SALSA: Sacramento Area Latino Study on Aging;

a Model 1: unadjusted;

b Model 2: age, gender, education;

c Model 3: age, gender, education, diabetes, atrial fibrillation, smoking, coronary heart disease/PAD, BMI, hypertension, and hyperlipidemia.

## Discussion

Using data from an elderly cohort of Latinos residing in Sacramento County, California, we found a significant association between IgG antibody levels to *H. pylori* and incident stroke after adjusting for socio-demographics and stroke risk factors. In contrast, we found no association between sero-positivity or IgG antibody levels to CMV, HSV1, *T. gondii*, or VZV and risk of stroke.

Our finding of null associations between sero-positivity to the five persistent pathogens and incident stroke are in agreement with existing prospective studies [13], [14], [15]. In contrast to these studies based on sero-status, our findings pertaining to antibody levels to *H. pylori* and stroke risk may be the result of our ability to more accurately assess the effect of exposure to this pathogen, given the evidence that antibody level may be a more consistent predictor of inflammatory outcomes [17]. It is unclear why *H. pylori* alone was associated with incident stroke. However, there are biologically plausible pathways by which *H. pylori* may influence incident stroke. For instance, lipopolysaccharides on the outer membrane of *H. pylori* have been reported to bind to Toll-like receptors on endothelial, monocyte and macrophage cells, which results in endothelial damage and the initiation of atherosclerosis [25]. Recently, *H. pylori* was reported to increase diabetes risk in the SALSA cohort [26]. However, when we adjusted for diabetes status in our analysis, the ORs were not significantly attenuated, suggesting that diabetes may not substantially mediate the *H. pylori*-stroke association. Another possible pathway linking *H. pylori* to incident stroke is through folic acid and Vitamin B12, both of which are decreased with exposure to this pathogen, which results in increased serum plasma homocysteine, a known risk factor for cardiovascular disease and stroke [27], [28], [29], [30], [31], [32].

An important link was recently made between sero-positivity to *H. pylori* and incident dementia, which is most commonly caused by Alzheimer’s disease [33]. Several mechanisms could explain this reported association, including the accumulation of risk factors for cardiovascular disease with chronic *H. pylori* exposure in the elderly, which could increase both risk of developing dementia as well as cognitive decline [33]. The previously discussed results, and those of the current study, offer added support for the extra-gastric effects of *H. pylori*, particularly among the elderly, and links two vascular brain disorders to this particular persistent pathogen. Future studies are needed to confirm the association between *H. pylori* and stroke risk. If our association is confirmed, investigations into possible mechanisms underlying this association are warranted.

In terms of our hypothesis that pathogen burden increases stroke risk, our fully adjusted models did not support this relationship. However, our null results are in agreement with the majority of the existing studies [13], [16]. On the other hand, Elkind et al. reported a significant positive association between a weighted index of pathogen burden (including *H. pylori*, C. pneumoniae, CMV, HSV 1 and 2) and incident stroke (HR: 1.39, 95% CI: 1.02–1.90) [14]. The infectious disease burden index used in our analysis was based on summed parameter estimates from models containing individual sero-positive pathogen exposures, which does not account for the potential variability in the strength of the association between each persistent pathogen and stroke risk. It is important to note, however, that comparisons across studies with different study populations, exposure and outcome definitions, as well as variables included in multivariable adjustment, should be done with caution.

Given our result of a significant association between pathogen burden defined as sum of the number of pathogens producing a high antibody level and stroke risk that was attenuated from unadjusted to fully-adjusted models, it is possible that one or more of our adjustment variables lies on the causal pathway. For example, inflammation may partially explain the association between pathogen burden and incident stroke. Inflammatory cytokines are associated with cerebral blood vessel constriction [34], [35], [36], which links inflammation to hypertension, a predominant modifiable risk factor for stroke [37], [38], [39], [40], [41]. Furthermore, pathogen burden, including sero-prevalence to C. pneumoniae, Mycoplasma pneumoniae, *H. pylori* and Coxsackie virus, have been associated with hypertension [42]. Given these data, the idea that exposure to more than one pathogen increases systemic inflammation and hypertension risk, thereby increasing incident stroke, is conceivable. The development of diabetes mellitus is also associated with inflammatory markers [43], [44], [45]. The inflammatory pathway that is induced by pathogen burden could increase diabetes risk, especially given the recent report of an association between *H. pylori* and incident diabetes in the SALSA cohort [26].

Our study has several strengths. To our knowledge, our study is the first to use prospectively collected, population-based data to investigate the link between IgG antibody levels to persistent pathogens and stroke risk in MAs. We used discrete-time regression, which may have permitted us to more accurately assess the time-varying effect of persistent pathogen exposure on stroke risk, and to account for temporal changes in risk factors. We also operationalized pathogen burden in several different ways, which has not been done in the stroke literature. Finally, our research focused specifically on MAs, a group disproportionately affected by persistent pathogens and stroke [3].

The following limitations of the current study should be considered. First, our primary endpoint, incident stroke, was largely based on self-report. However, sensitivity rates for self-reported stroke ranging from 80–98% have been reported in the elderly [46], [47]. Any misclassification in the outcome would likely bias our results toward the null, since self-reported stroke is unlikely to be associated with exposure to persistent pathogens. However, it is possible that individuals with infections caused by *H. pylori* are more likely to seek medical attention, and as a result, may be more likely to have mild strokes diagnosed. Nevertheless, it is likely that most *H. pylori* infections are undiagnosed in this population since a very small percentage of participants were treated with a proton pump inhibitor and/or antibiotics compatible with *H. pylori* infection over the study period [26]. It is possible that those exposed to H. pylori may shun aspirin, thereby increasing their risk of stroke, however, we were unable to account for the use of immune altering or anti-inflammatory medications in our multivariate models, due to a substantial number of incomplete drug reports during follow-up. Given the likelihood that medication usage changes with time, we could not justify extrapolating these data. However, we found no difference in the baseline distribution of aspirin use by *H. pylori* serostatus (χ^2^ p-value: 0.8565). Next, we could not distinguish between stroke types, which may bias our associations towards the null, as infection is more likely to be associated with ischemic stroke through atherosclerotic mechanisms. Further, additional pathogens, such as C. pneumoniae and periodontal pathogens, which have been previously studied for their links to stroke, were not available [48], [49], [50], [51], [52]. Also, apolipoprotein E is involved in cellular cholesterol and lipid packaging [53] and has been reported to affect the outcome of many persistent pathogens [54], [55], [56], [57], [58], [59]. In the SALSA cohort, the prevalence of Apolipoprotein E is13.4 percent. While APOE4 is not directly related to our outcome of interest, it may be an important factor in future studies aimed at understanding the effect of persistent pathogens on chronic diseases. With our risk factor and pathogen carry-forward methods, we assume that this information is constant, which may not be true for all individuals, however, our correlation analysis provided justification for this analytic approach. Furthermore, in a post-hoc sensitivity analysis, we found no significant associations between baseline pathogen exposures and incident stroke during follow-up. However, we would contend that our approach of using baseline pathogen data and updating it with longitudinal data as available, allows us to more accurately estimate the effect of antibody levels to persistent pathogen on stroke risk, compared to using baseline antibody levels only. Next, we assumed biologic interaction between the persistent pathogens was absent when we defined pathogen burden, which may not be true. It is possible that there are additive, supra or sub-additive interactions between pathogens [60]. Our study may have been under-powered to detect a significant associations between individual persistent pathogens, pathogen burden and incident stroke because of the relatively low number of stroke cases over the study period. Lastly, the generalizability of our study results is limited to elderly MAs. Nevertheless, identification of novel risk factors in MAs is particularly important given the growth of this aging population in the US.

In summary, we found a significant association between antibody levels to *H. pylori* and incident stroke among MAs. Null associations were found for the other pathogens studied as well as pathogen burden and incident stroke. Future studies are needed to confirm our results.

## Supporting Information

File S1 **Supporting tables. Table S1**. **Correlations of CMV serostatus by visit, SALSA, California, 1998–2008.** BL: baseline; FV3–6: follow-up visit 3–6; CMV: cytomegalovirus; SALSA: Sacramento Area Latino Study on Aging; ^a^Asymmetric; ^b^Symmetric. **Table S2. Variability of CMV Immunoglobulin G antibody levels by visit, SALSA, California, 1998–2008.** SALSA: Sacramento Area Latino Study on Aging; CMV: cytomegalovirus. **Table S3. Correlations of HSV1 serostatus by visit, SALSA, California, 1998–2008.** BL: baseline; FV3–6: follow-up visit 3–6; HSV1: Herpes Simplex Virus type 1; ^a^Asymmetric; ^b^Symmetric; Gamma: range (−1, 1); Kappa interpretation: (0.0< Kappa >0.4 = marginal agreement, 0.4< kappa >0.75 = good agreement, Kappa >0.75: excellent agreement; Somers D: range (−1,1); Lambda (asymmetric and symmetric): range (0,1). **Table S4. Variability of HSV1 Immunoglobulin G antibody levels by visit, SALSA, California, 1998–2008.** HSV1: Herpes simplex virus type 1; SALSA: Sacramento Area Latino Study on Aging. **Table S5. Correlations of** ***H. Pylori*** **serostatus by visit, SALSA, California, 1998–2008.** BL, baseline; FV3–6: follow-up visit 3–6; *H. pylori: Helicobacter Pylori*; ^a^Asymmetric; ^b^Symmetric Gamma: range (−1, 1); Kappa interpretation: (0.0< Kappa >0.4 = marginal agreement, 0.4< kappa >0.75 = good agreement, Kappa >0.75: excellent agreement; Somers D: range (−1,1); Lambda (asymmetric and symmetric): range (0,1); SALSA: Sacramento Area Latino Study on Aging. **Table S6. Variability of** ***H. Pylori*** **Immunoglobulin G antibody level by visit, SALSA, California, 1998–2008.** *H. Pylori: Helicobacter pylori;* SALSA: Sacramento Area Latino Study on Aging. **Table S7. Correlations of VZV serostatus by visit, SALSA, California, 1998–2008.** BL: baseline; FV3–6: follow-up visit 3–6;VZV, Varicella Zoster Virus; ^a^Asymmetric; ^b^Symmetric; Gamma: range (−1, 1); Kappa interpretation: (0.0< Kappa >0.4 = marginal agreement, 0.4< kappa >0.75 = good agreement, Kappa >0.75: excellent agreement; Somers D: range (−1,1); Lambda (asymmetric and symmetric): range (0,1); SALSA: Sacramento Area Latino Study on Aging. **Table S8. Variability of VZV Immunoglobulin G antibody levels by SALSA, California, 1998–2008.** VZV: varicella zoster virus; SALSA: Sacramento Area Latino Study on Aging. **Table S9.** ***T. gondii*** **serostatus correlations by visit, SALSA, California, 1998–2008.** BL: baseline; FV3–6: follow-up visit 3–6*; T. gondii: Toxoplasma gondii*; ^a^Asymmetric; ^b^Symmetric; Gamma: range (−1, 1); Kappa interpretation: (0.0< Kappa >0.4 = marginal agreement, 0.4< kappa >0.75 = good agreement, Kappa >0.75: excellent agreement; Somers D: range (−1,1); Lambda (asymmetric and symmetric): range (0,1); SALSA: Sacramento Area Latino Study on Aging. **Table S10. Variability of** ***T. gondii*** **Immunoglobulin G antibody levels by visit SALSA, California, 1998–2008.** *T. gondii*: *Toxoplasma gondii*; SALSA: Sacramento Area Latino Study on Aging.
(DOCX)
