# Supplementary material for: Antibody Levels to Persistent Pathogens and Incident Stroke in Mexican Americans
Source: PLoS One. 2013 Jun 14;8(6):e65959. doi: 10.1371/journal.pone.0065959 (PMC3682951; doi:10.1371/journal.pone.0065959)
Supplement: File S1 — Supporting tables. Table S1. Correlations of CMV serostatus by visit, SALSA, California, 1998–2008. BL: baseline; FV3–6: follow-up visit 3–6; CMV: cytomegalovirus; SALSA: Sacramento Area Latino Study on Aging; aAsymmetric; bSymmetric. Table S2. Variability of CMV Immunoglobulin G antibody levels by visit, SALSA, California, 1998–2008. SALSA: Sacramento Area Latino Study on Aging; CMV: cytomegalovirus. Table S3. Correlations of HSV1 serostatus by visit, SALSA, California, 1998–2008. BL: baseline; FV3–6: follow-up visit 3–6; HSV1: Herpes Simplex Virus type 1; aAsymmetric; bSymmetric; Gamma: range (−1, 1); Kappa interpretation: (0.0< Kappa >0.4 = marginal agreement, 0.4< kappa >0.75 = good agreement, Kappa >0.75: excellent agreement; Somers D: range (−1,1); Lambda (asymmetric and symmetric): range (0,1). Table S4. Variability of HSV1 Immunoglobulin G antibody levels by visit, SALSA, California, 1998–2008. HSV1: Herpes simplex virus type 1; SALSA: Sacramento Area Latino Study on Aging. Table S5. Correlations of H. Pylori serostatus by visit, SALSA, California, 1998–2008. BL, baseline; FV3–6: follow-up visit 3–6; H. pylori: Helicobacter Pylori; aAsymmetric; bSymmetric Gamma: range (−1, 1); Kappa interpretation: (0.0< Kappa >0.4 = marginal agreement, 0.4< kappa >0.75 = good agreement, Kappa >0.75: excellent agreement; Somers D: range (−1,1); Lambda (asymmetric and symmetric): range (0,1); SALSA: Sacramento Area Latino Study on Aging. Table S6. Variability of H. Pylori Immunoglobulin G antibody level by visit, SALSA, California, 1998–2008. H. Pylori: Helicobacter pylori; SALSA: Sacramento Area Latino Study on Aging. Table S7. Correlations of VZV serostatus by visit, SALSA, California, 1998–2008. BL: baseline; FV3–6: follow-up visit 3–6;VZV, Varicella Zoster Virus; aAsymmetric; bSymmetric; Gamma: range (−1, 1); Kappa interpretation: (0.0< Kappa >0.4 = marginal agreement, 0.4< kappa >0.75 = good agreement, Kappa >0.75: excellent agreement; Somers D: range (−1,1); Lambda (asy [file pone.0065959.s001.docx]

**Table S1**. Correlations of CMV serostatus by visit, SALSA, California, 1998-2008.

|  | **BL vs. FV3** | **BL vs. FV4** | **BL vs. FV5** | **BL vs. FV6** | **FV3 vs. FV4** | **FV5 vs. Fv6** |
| --- | --- | --- | --- | --- | --- | --- |
| Gamma | 0.9157 | 0.8754 | 0.7832 | 0.8406 | 0.9147 | 0.8846 |
| Kappa | 0.6662 | 0.6002 | 0.5397 | 0.5615 | 0.6054 | 0.6798 |
| Somers D | 0.6739 | 0.6129 | 0.5316 | 0.5614 | 0.7426 | 0.6872 |
| Lambda^a^ | 0.4464 | 0.4000 | 0.4211 | 0.4051 | 0.4737 | 0.4571 |
| Lambda^b^ | 0.4359 | 0.4043 | 0.4026 | 0.3757 | 0.4444 | 0.4507 |
| McNemar’s p- value | 0.4168 | 0.2378 | 0.7851 | 0.0644 | 0.5319 | 0.9643 |

BL: baseline; FV3-6: follow-up visit 3-6; CMV: cytomegalovirus; SALSA: Sacramento Area Latino Study on Aging; ^a^Asymmetric; ^b^Symmetric

**Table S2**. Variability of CMV Immunoglobulin G antibody levels by visit, SALSA, California, 1998-2008.

|  | **Intra-class correlation coefficient** | **Coefficient of Variation** |
| --- | --- | --- |
| Baseline | 0.5135 | 48.5461 |
| Follow-up visit 3 | 0.5228 | 46.3835 |
| Follow-up visit 4 | 0.5380 | 49.0630 |
| Follow-up visit 5 | 0.5146 | 47.2572 |
| Follow-up visit 6 | 0.5500 | 47.8570 |

SALSA: Sacramento Area Latino Study on Aging; CMV: cytomegalovirus

**Table S3**. Correlations of HSV1 serostatus by visit, SALSA, California, 1998-2008.

|  | **BL vs. FV3** | **BL vs. FV4** | **BL vs. FV5** | **BL vs. FV6** | **FV3 vs. FV4** | **FV5 vs. FV6** |
| --- | --- | --- | --- | --- | --- | --- |
| Gamma | 0.9323 | 0.9159 | 0.9450 | 0.9452 | 0.9496 | 0.8870 |
| Kappa | 0.7008 | 0.6136 | 0.6002 | 0.6992 | 0.7967 | 0.6354 |
| Somers D | 0.7236 | 0.6692 | 0.7209 | 0.7372 | 0.8432 | 0.7036 |
| Lambda^a^ | 0.5102 | 0.3704 | 0.5185 | 0.4923 | 0.6842 | 0.5172 |
| Lambda^b^ | 0.5049 | 0.3860 | 0.4310 | 0.4929 | 0.6842 | 0.5345 |
| McNemar’s p- value | 0.5044 | 0.3387 | 0.1059 | 0.0785 | 0.9536 | 0.4980 |

BL: baseline; FV3-6: follow-up visit 3-6; HSV1: Herpes Simplex Virus type 1; ^a^Asymmetric; ^b^Symmetric; Gamma: range (-1, 1); Kappa interpretation: (0.0< Kappa >0.4= marginal agreement, 0.4< kappa >0.75= good agreement, Kappa >0.75: excellent agreement; Somers D: range (-1,1); Lambda (asymmetric and symmetric): range (0,1)

**Table S4**. Variability of HSV1 Immunoglobulin G antibody levels by visit, SALSA, California, 1998-2008.

|  | **Intra-class correlation coefficient** | **Coefficient of Variation** |
| --- | --- | --- |
| Baseline | 0.6065 | 51.0447 |
| Follow-up visit 3 | 0.6324 | 51.7672 |
| Follow-up visit 4 | 0.5970 | 51.0291 |
| Follow-up visit 5 | 0.6630 | 56.9176 |
| Follow-up visit 6 | 0.6490 | 51.4561 |

HSV1: Herpes simplex virus type 1; SALSA: Sacramento Area Latino Study on Aging

**Table S5**. Correlations of *H. Pylori* serostatus by visit, SALSA, California, 1998-2008.

|  | **BL vs. FV3** | **BL vs. FV4** | **BL vs. FV5** | **BL vs. FV6** | **FV3 vs. FV4** | **FV5 vs. FV6** |
| --- | --- | --- | --- | --- | --- | --- |
| Gamma | 0.8920 | 0.8829 | 0.7980 | 0.9190 | 0.9230 | 0.8722 |
| Kappa | 0.5873 | 0.4444 | 0.4081 | 0.4820 | 0.7159 | 0.5783 |
| Somers D | 0.6379 | 0.6305 | 0.5565 | 0.7324 | 0.7581 | 0.6369 |
| Lambda^a^ | 0.3462 | 0.1923 | 0.2308 | 0.2500 | 0.5714 | 0.3939 |
| Lambda^b^ | 0.3163 | 0.1538 | 0.1515 | 0.1527 | 0.5641 | 0.3731 |
| McNemar’s p- value | 0.2867 | 0.1038 | 0.0906 | 0.0002 | 0.5319 | 0.7932 |

BL, baseline; FV3-6: follow-up visit 3-6; *H. pylori: Helicobacter Pylori*; ^a^Asymmetric; ^b^Symmetric Gamma: range (-1, 1); Kappa interpretation: ( 0.0< Kappa >0.4= marginal agreement, 0.4< kappa >0.75= good agreement, Kappa >0.75: excellent agreement; Somers D: range (-1,1); Lambda (asymmetric and symmetric): range (0,1); SALSA: Sacramento Area Latino Study on Aging

**Table S6**. Variability of *H. Pylori* Immunoglobulin G antibody level by visit, SALSA, California, 1998-2008.

|  | **Intra-class correlation coefficient** | **Coefficient of Variation** |
| --- | --- | --- |
| Baseline | 0.7830 | 50.7843 |
| Follow-up visit 3 | 0.7857 | 57.0787 |
| Follow-up visit 4 | 0.8016 | 59.2287 |
| Follow-up visit 5 | 0.7958 | 61.8262 |
| Follow-up visit 6 | 0.7958 | 61.0098 |

*H. Pylori: Helicobacter pylori;* SALSA: Sacramento Area Latino Study on Aging

**Table S7**. Correlations of VZV serostatus by visit, SALSA, California, 1998-2008.

|  | **BL vs. FV3** | **BL vs. FV4** | **BL vs. FV5** | **BL vs. FV6** | **FV3 vs. FV4** | **FV5 vs. FV6** |
| --- | --- | --- | --- | --- | --- | --- |
| Gamma | 0.4446 | 0.3900 | 0.4011 | 0.4315 | 0.4147 | 0.5309 |
| Kappa | 0.5492 | 0.4741 | 0.4292 | 0.4678 | 0.5460 | 0.5856 |
| Somers D | 0.3537 | 0.2824 | 0.2960 | 0.3117 | 0.3154 | 0.4192 |
| Lambda^a^ | 0.5333 | 0.3812 | 0.3697 | 0.4452 | 0.5541 | 0.5556 |
| Lambda^b^ | 0.4956 | 0.3571 | 0.3451 | 0.3779 | 0.5484 | 0.5200 |
| McNemar’s p- value | 0.0008 | 0.0010 | 0.1701 | <0.0001 | 0.2896 | 0.1069 |

BL: baseline; FV3-6: follow-up visit 3-6;VZV, Varicella Zoster Virus; ^a^Asymmetric; ^b^Symmetric; Gamma: range (-1, 1); Kappa interpretation: ( 0.0< Kappa >0.4= marginal agreement, 0.4< kappa >0.75= good agreement, Kappa >0.75: excellent agreement; Somers D: range (-1,1); Lambda (asymmetric and symmetric): range (0,1); SALSA: Sacramento Area Latino Study on Aging

**Table S8**. Variability of VZV Immunoglobulin G antibody levels by SALSA, California, 1998-2008.

|  | **Intra-class correlation coefficient** | **Coefficient of Variation** |
| --- | --- | --- |
| Baseline | 0.1903 | 52.6606 |
| Follow-up visit 3 | 0.2278 | 52.1039 |
| Follow-up visit 4 | 0.1990 | 51.5351 |
| Follow-up visit 5 | 0.2199 | 54.0146 |
| Follow-up visit 6 | 0.2331 | 52.4651 |

VZV: varicella zoster virus; SALSA: Sacramento Area Latino Study on Aging

|  | **BL vs. FV3** | **BL vs. FV4** | **BL vs. FV5** | **BL vs. FV6** | **FV3 vs. FV4** | **FV5 vs. FV6** |
| --- | --- | --- | --- | --- | --- | --- |
| Gamma | 0.8387 | 0.8725 | 0.6834 | 0.8001 | 0.8613 | 0.6993 |
| Kappa | 0.7711 | 0.7913 | 0.6568 | 0.7118 | 0.7475 | 0.7810 |
| Somers D | 0.7143 | 0.7495 | 0.5029 | 0.6423 | 0.7077 | 0.6342 |
| Lambda^a^ | 0.6988 | 0.7624 | 0.5429 | 0.6505 | 0.7536 | 0.7143 |
| Lambda^b^ | 0.7069 | 0.7636 | 0.5789 | 0.6490 | 0.7500 | 0.7007 |
| McNemar’s p- value | 0.0587 | 0.3973 | 0.1321 | 0.4461 | 0.0937 | 0.1386 |

**Table S9**. *T. gondii* serostatus correlations by visit, SALSA, California, 1998-2008.

BL: baseline; FV3-6: follow-up visit 3-6*; T. gondii: Toxoplasma gondii*; ^a^Asymmetric; ^b^Symmetric; Gamma: range (-1, 1); Kappa interpretation: ( 0.0< Kappa >0.4= marginal agreement, 0.4< kappa >0.75= good agreement, Kappa >0.75: excellent agreement; Somers D: range (-1,1); Lambda (asymmetric and symmetric): range (0,1); SALSA: Sacramento Area Latino Study on Aging

**Table S10**. Variability of *T. gondii* Immunoglobulin G antibody levels by visit SALSA, California, 1998-2008.

|  | **Intra-class correlation coefficient** | **Coefficient of Variation** |
| --- | --- | --- |
| Baseline | 0.5957 | 99.0583 |
| Follow-up visit 3 | 0.6174 | 103.1960 |
| Follow-up visit 4 | 0.6016 | 100.8542 |
| Follow-up visit 5 | 0.5783 | 111.7730 |
| Follow-up visit 6 | 0.6011 | 100.5119 |

*T. gondii*: *Toxoplasma gondii*; SALSA: Sacramento Area Latino Study on Aging
